# Impaired glucose metabolism in patients with diabetes, prediabetes, and obesity is associated with severe COVID‐19

**DOI:** 10.1002/jmv.26227

**Published:** 2020-07-17

**Authors:** Stephen M. Smith, Avinash Boppana, Julie A. Traupman, Enrique Unson, Daniel A. Maddock, Kathy Chao, David P. Dobesh, Adam Brufsky, Ruth I. Connor

**Affiliations:** ^1^ The Smith Center for Infectious Diseases and Urban Health East Orange New Jersey; ^2^ Saint Barnabas Medical Center RWJBarnabas Health Livingston New Jersey; ^3^ UPMC Hillman Cancer Center, Magee‐Women's Hospital University of Pittsburgh Pittsburgh Pennsylvania; ^4^ Dartmouth‐Hitchcock Medical Center Lebanon New Hampshire

**Keywords:** COVID‐19, diabetes mellitus, glucose metabolism disorder, hyperglycemia, obesity, SARS‐CoV‐2

## Abstract

Background

Identification of risk factors of severe coronavirus disease 2019 (COVID‐19) is critical for improving therapies and understanding severe acute respiratory syndrome coronavirus 2 (SARS‐CoV‐2) pathogenesis. We analyzed 184 patients hospitalized for COVID‐19 in Livingston, New Jersey for clinical characteristics associated with severe disease. The majority of patients with COVID‐19 had diabetes mellitus (DM) (62.0%), Pre‐DM (23.9%) with elevated fasting blood glucose (FBG), or a body mass index >30 with normal hemoglobin A1c (HbA1C) (4.3%). SARS‐CoV‐2 infection was associated with new and persistent hyperglycemia in 29 patients, including several with normal HbA1C levels. Forty‐four patients required intubation, which occurred significantly more often in patients with DM as compared with non‐diabetics. Severe COVID‐19 occurs in the presence of impaired glucose metabolism in patients, including those with DM, preDM, and obesity. COVID‐19 is associated with elevated FBG and several patients presented with new onset DM or in DKA. The association of dysregulated glucose metabolism and severe COVID‐19 suggests that SARS‐CoV‐2 pathogenesis involves a novel interplay with glucose metabolism. Exploration of pathways by which SARS‐CoV‐2 interacts glucose metabolism is critical for understanding disease pathogenesis and developing therapies.

## Highlights

- The vast majority of hospitalized COVID‐19 patients had diabetes mellitus (60.2%), prediabetes (23.9%) or obesity alone (4.3%).
- All COVID‐19 patients requiring intubation were diabetic, obese or both.
- SARS‐CoV‐2 infection is associated with new and persistent hyperglycemia in non‐diabetics, consistent with new‐onset diabetes, and with diabetic‐ketoacidosis in diabetics.
- At presentation, a patient s diabetes status, BMI, A1C level and initial blood glucose level, along with age, can be used to identify patients at‐risk for severe COVID‐19.
- Through a novel pathogenic interaction with glucose metabolism, SARS‐CoV‐2 worsens hyperglycemia, which, in turn, worsens severity of COVID‐19 pneumonia.
- Elucidation and understanding this unique interaction between virus and host could lead to COVID‐19 therapeutic strategies, which target glucose metabolism alone.

## 1 INTRODUCTION

Severe acute respiratory syndrome coronavirus 2 (SARS‐CoV‐2), the causative agent of coronavirus disease 2019 (COVID‐19) was first detected in late December 2019 in Wuhan province in China and rapidly spread across the globe.^1^ To date over 8.1 million cases of COVID‐19 have been reported worldwide with approximately 2.1 million cases in the United States and 116 862 deaths.^2^, ^3^

Early reports from China and later Italy examined risk factors for severe COVID‐19 and identified advanced age as a major indicator for increased mortality.^4^, ^5^ A recent study of over 4000 patients with confirmed COVID‐19 in the United States found older age (>65 years), obesity (body mass index [BMI] > 40), chronic kidney disease and a history of heart failure were most associated with hospitalization, while critical illness was linked to low oxygen saturation (<88%) at admission, first d‐dimer (>2500), first ferritin (>2500), and first C‐reactive protein (>200) indicating hypoxia and inflammation in patients with clinically progressive disease.^6^

A number of studies have identified an increased risk of severe disease in COVID‐19 patients with underlying health conditions. Data compiled by the COVID‐19 Associated Hospitalization Surveillance Network identified hypertension (49.7%), obesity (48.3%), chronic lung disease (34.6%), diabetes mellitus (DM) (28.3%), and cardiovascular disease (27.8%) as the most commonly found co‐morbidities among hospitalized patients with COVID‐19 in the United States.^3^ A recent study of Lopinavir‐Ritonavir in adults hospitalized with severe COVID‐19 found 13% of patients had DM, reinforcing early observations that diabetes is a risk factor for more severe disease.^7^ This is supported by data from a study of 24 patients hospitalized for COVID‐19 in nine Seattle‐area hospitals in which 58% of critically ill patients had DM and an average BMI of 33.^8^ Interestingly, in the 2003 SARS‐CoV outbreak in China, hyperglycemia and DM were also noted as risk factors for mortality and morbidity.^9^ These observations and several in‐depth reviews^10^, ^11^, ^12^ have raised concerns that diabetics with elevated fasting blood glucose are at increased risk of developing severe COVID‐19.

We report here our experience of 184 patients admitted for COVID‐19 to a teaching hospital in Livingston, New Jersey within the epicenter of the SARS‐Cov‐2 pandemic in the United States. Extending early observations, we find the vast majority of our patients with COVID‐19 are diabetic, prediabetic, or obese. Moreover, we identify COVID‐19 patients with prediabetes (preDM) and others with normal hemoglobin A1c (HbA1C) levels who developed new onset DM, similar in presentation to type 1 DM, coincident with recent acquisition of SARS‐CoV‐2 infection. Our data establish that impaired glucose metabolism, due to either DM or obesity, is significantly associated with severe COVID‐19 in this high‐risk population.

## 2 METHODS

### 2.1 Study population

Patients with COVID‐19 were referred to our practice by the emergency medicine department or admitting physician at a large suburban hospital (Saint Barnabas Medical Center, Livingston, New Jersey). Consecutive admission of 184 patients with COVID‐19 occurred over a period of 7 weeks (16 March‐2 May 2020) and all patients received care through our practice. A diagnosis of COVID‐19 was made on the basis of a confirmed positive laboratory test for SARS‐CoV‐2 in 177 patients. The remaining patients were diagnosed based on clinical presentation including new onset hypoxia, increased lactate dehydrogenase, increased D‐dimer, increased ferritin, and elevated blood glucose. All patients were included in the analysis for this study. Ethical approval for the study was granted by the Institutional Review Board of St. Barnabas Medical Center.

### 2.2 Diabetes status

A high percentage of patients testing positive for SARS‐CoV‐2 and referred to our practice were already known diabetics and receiving treatment for DM at the time of admission. We used the American Diabetes Association definitions to diagnose DM, new onset DM and preDM.^13^ A new diagnosis of DM was made in patients previously unaware of their condition based on an HbA1C > 6.4%. New onset DM was defined by persistently elevated fasting blood glucose (FBG) > 125 mg/dL and requiring insulin therapy. PreDM was defined by an HbA1C of 5.7% to 6.4%. Nondiabetic patients were defined as having an HbA1C < 5.7% and FBG ≤ 125 mg/dL. Fever was defined as Tmax ≥100°F during the first 6 hours after admission. Hypoxia was defined as room oxygen saturation <94%.

### 2.3 Outcomes

The primary indicator of severe COVID‐19 was intubation. The need for intubation was determined on the basis of clinical presentation in patients receiving full care throughout their hospitalization. Death during hospitalization included patients put on comfort care at any time during or after admission. Comfort care measures were determined by the primary attending physician and included but were not limited to morphine drips or intensive care without further escalation of care. We developed a simple, scoring system for outcomes, based up a patient's diabetes status, BMI, A1C, Age, and initial blood glucose level. For details, please see Supplemental Information.

### 2.4 Statistical analyses

A one‐sample proportion *Z*‐test was used to determine the prevalence of DM, preDM, and nonDM in patients with COVID‐19 as compared with the US population. The sample size used for this analysis was 184 with at least 10 patients in each DM status. One‐sided hypothesis tests were used to determine if the proportions of COVID‐19 patients with DM and preDM were larger than the U.S. population proportions, and if the proportion of NonDM patients was smaller than the U.S. population proportion. A *χ* ^2^ test was used to determine significance between intubation and diabetes status within each patient group. 95% confidence intervals were calculated using standard errors. Statistical significance was defined as a *P *< .05. All statistical analyses were performed using R version 3.4.4.

## 3 RESULTS

### 3.1 Demographic and clinical characteristics of the patients

During a seven‐week period, 184 patients were admitted to the hospital for COVID‐19 and referred to our practice. The average age of study patients was 64.4 years (range, 21‐100 years.) with 86 (46.7%) females and 98 (53.3%) males (Table 1). The racial and ethnic composition of the study population was black (53.8%), white (25.5%), Latino (6.5%), and Asian (6.0%). Clinical presentation at the time of admission included hypoxia (83.7%) and fever (62.5%) (Table 1). Hypoxia and fever occurred together often (48.9%); only a small percentage (7.6%) of patients presented without fever or hypoxia. In addition to DM, as described herein, the most common pre‐existing conditions included hypertension (60.3%), hyperlipidemia (33.7%), dementia (13.0%), chronic kidney disease (13.0%), coronary artery disease (12.0%), and congestive heart failure (10.9%) (Table 1).

**Table 1:** Demographic and clinical characteristics of patients with COVID‐19

Characteristic	Patients
Age	N = 184
Avg, y	64.4 (21‐100)
Age, no. (%)	
≤60	75 (40.8)
>60	109 (59.2)
Sex, no. (%)	
Female	86 (46.7)
Male	98 (53.3)
Race and ethnicity, no. (%)	
Black	99 (53.8)
White	47 (25.5)
Latino	12 (6.5)
Asian	11 (6.0)
Other	15 (8.2)
Presentation values	
BMI, Avg (range)	29.8 (17.5‐61.4)
HbA1C, Avg % (range)	7.4 (4.1‐14.7)
Glucose, Avg mg/dL (range)	179.9 (11‐1129)
T ≥ 100°F, no. (%)	115 (62.5)
Hypoxia on room air, no. (%)	154 (83.7)
Pre‐existing conditions, no (%)	
Hypertension	111 (60.3%)
Hyperlipidemia	62 (33.7%)
Dementia	24 (13.0%)
Chronic kidney disease	24 (13.0%)
Coronary artery disease	22 (12.0%)
Congestive heart failure	20 (10.9%)
Asthma	18 (9.8%)
Atrial fibrillation	14 (7.6%)
Cancer	17 (9.2%)
COPD	12 (6.5%)
Cerebrovascular accident	10 (5.4%)
Transplant, renal	5 (2.7%)

Abbreviations: Avg, Average; BMI, body mass index; COPD, chronic obstructive pulmonary disease; COVID‐19, coronavirus disease 2019; HbA1C, hemoglobin A1c.

### 3.2 Increased prevalence of diabetes, pre‐diabetes, and obesity

The majority of patients with COVID‐19 had DM (62.0%), preDM (23.9%) or BMI > 30 with normal HbA1C (4.3%). The prevalence of DM was 4.7‐fold higher in this patient group as compared with the general US population, while the prevalence of preDM was 1.3‐fold higher.^14^ A significant number of patients were clinically obese. The mean BMI of the study patients was 29.8 (17.5‐61.4), including 20 patients with BMIs > 40. HbA1C levels measured at admission in 171 patients also showed significant elevation with 64 patients (37.4%) having values between 5.7% and 6.4% and 82 (48.0%) having values ≥6.5%.

### 3.3 Age relationship to BMI, HbA1c, and initial blood glucose level

To determine whether patient age was associated with differences in clinical presentation, data on BMI, HbA1C, and initial FBG were stratified by age at admission. The rates of DM and preDM were similar in patients ≤60 years as compared with those >60 years (Table 2) as were mean initial FBG levels (200.5 vs 165.4 mg/dL). However, patients ≤60 years of age were significantly more likely to be clinically obese. As compared with patients >60 years, the frequency of obesity and the mean BMI in those ≤60 years were significantly higher (26.6% vs 65.3% and 27.2 vs 33.4, respectively; *P* < .0001) (Table 3). Patients ≤60 years were also significantly more likely to be severely obsese with a BMI > 40 (20.0% vs 3.7%; *P* = .0013). Similarly, patients ≤60 years had a significantly higher mean HbA1C level than older patients (8.0% vs 6.9%; *P* = .003) suggesting more pronounced metabolic dysregulation in younger patients. Taken together, these data indicate that younger patients may be more likely to present with abnormalities in glucose metabolism due to obesity, which may put them at increased risk of developing severe COVID‐19. These findings are consistent with a recent report of 265 patients with COVID‐19 demonstrating a significant inverse correlation of age and BMI in which younger patients hospitalized for COVID‐19 were more often obese.^15^

**Table 2:** Diabetes status, obesity rate, and HbA1C levels by age

	Patients ≤60 y (N = 75)	Patients >60 y (N = 109)	*P* value
DM (%)	64.0	60.6	.38
PreDM (%)	24.0	23.9	.50
NonDM (%)	12.0	15.6	.32
Mean FBG, mg/dL	200.5	165.4	.10
Mean BMI	33.4	27.2	<.0001
BMI > 30 (%)	65.3	26.6	<.0001
BMI > 40 (%)	20.0	3.7	.0004
HbA1C (%)	8.0	6.9	.003

Abbreviations: BMI, body mass index; DM, diabetes mellitus; FBG, fasting blood glucose; HbA1C, hemoglobin A1c; nonDM, nondiabetic; preDM, pre‐diabetes.

**Table 3:** Hyperglycemia and obesity in patients with COVID‐19 requiring intubation

	Intubated	Not intubated	*P* value (95% CI)
N (%)	44 (23.9)	144 (76.1)	
Mean BMI	32.2	29.3	.030 (0.3‐5.8)
Mean HbA1C (%)	8.0	7.2	.034 (0.07‐1.67)
Mean FBG, mg/dL	238.0	163.7	.013 (9.02‐135.9)

Abbreviations: BMI, body mass index; CI, confidence interval; COVID‐19, coronavirus disease 2019; FBG, fasting blood glucose; HbA1C, hemoglobin A1c.

### 3.4 Association of obesity and uncontrolled glycemia with intubation

Intubation was evaluated as an indicator of COVID‐19 progression and severity in hospitalized patients. To determine whether higher rates of intubation were associated with uncontrolled glycemia, data on BMI, HbA1C, and FBS were evaluated for intubated patients and compared with their non‐intubated counterparts. Among 184 hospitalized patients receiving full care for COVID‐19, 44 (23.9%) required intubation. The mean BMI of patients requiring intubation was significantly higher than that of non‐intubated patients (32.3 vs 29.3; *P* = .030; 95% C.I. = 0.3‐5.8). More strikingly, patients with a BMI > 40 were intubated at a significantly higher rate than patients with BMIs < 25 (47.4 vs 15.6%; *P* = .0078).

HbA1C levels were available for 41 intubated patients and revealed only four (9.8%) had normal values. Of these, three were known to be diabetic and receiving treatment for DM. In total, 40 of 41 (97.6%) intubated patients had either elevated HbA1C or were receiving therapy for DM. As compared with patients not requiring intubation, the mean HbA1C of intubated patients was significantly higher (8.0 vs 7.2%; *P* = .034; confidence interval [CI] = 0.07‐1.67). Accordingly, the rate of intubation among patients with poorly‐controlled DM (HbA1C ≥ 7.5%) was significantly higher than that of patients with HbA1C < 7.5% (31.5% vs 17.8%; *P* = .045). The mean FBG at admission for intubated patients was also significantly increased when compared with that of patients not requiring intubation (238.0 vs 163.7 mg/dL; *P* = .013; CI = 9.02‐135.9) suggesting that uncontrolled glycemia, due to obesity or DM, is a significant risk factor for severe COVID‐19.

### 3.5 Intubation rate increases in non‐DM, preDM, and DM patients

To determine whether COVID‐19 severity was associated with diabetes status, patients requiring intubation were stratified as non‐DM, preDM, or DM. Determination of diabetic status was made on the basis of clinical presentation, HbA1C values and FBG levels for all patients. The majority (35 of 44; 79.5%) of intubated patients had DM, including seven newly diagnosed and five with new onset DM. Another seven (15.9%) were preDM with high FBG levels. Only one patient requiring intubation was non‐DM with normal HbA1C and FBG levels at admission, but was clinically obese with a BMI > 30.

Within the entire patient with COVID‐19 cohort, diabetes status was also associated with increasing rates of intubation. Among 25 patients with no diabetes and normal HbA1C levels, only one (4.0%) required intubation. Of patients with preDM, 18.5% (10 of 54) were intubated, while 28.85% (30 of 104) of patients with DM required intubation. Comparison of intubation rates demonstrated a significant difference between non‐DM patients (4.0%) and those with DM (difference = 24.85%; *P* = .0093: CI = 7.4%‐34.8%). These findings again suggest that changes in glycemia occurring with the onset and progression of diabetes are associated with a corresponding increase in the likelihood of severe COVID‐19 requiring intubation.

Twenty‐four patients died without intubation. The average age of these patients was 80.5 years (range, 45‐100 years) and the majority were put on comfort care with do not resuscitate orders in place. Among these patients 17 (70.8%) had DM, four (16.7%) were preDM, and three (12.5%) were nonDM.

We developed a simple scoring system, the Smith center COVID‐19 severity score, based upon age, diabetes status, BMI, A1C level, and initial blood glucose level. One hundred and sixty‐nine of the 184 patients had all needed measurements. For details see the supplemental data. One hundred and eleven of the 169 lived and were never intubated; 58 patients were intubated and/or expired; 44 patients expired (with or without prior intubation). The minimum and maximum total scores are 0 and 25. The mean scores for the alive, never intubated, intubated and/or expired, and expired groups were 10.2, 13.1, and 13.4, respectively. The differences between the alive/never intubated group and the other two groups were statistically significant with *P* < .0001. More importantly, 31 patients had a SCCSS ≤ 7, 30 had a good outcome; the other patient, who has end‐stage renal disease, was intubated and lived; none died. A screening system, such as the SCCSS, might be useful in triaging patients, especially in resource limited areas.

### 3.6 COVID‐19 associated hyperglycemia

Obesity, preDM and DM are typically associated with elevated blood glucose levels. We found blood glucose was increased in the majority of COVID‐19 patients at admission with a mean of 179.9 mg/dL. For most patients, these values were obtained after fasting. The presenting FBGs were markedly elevated in many patients with COVID‐19 and 15 (10.2%) had FBG levels >350 mg/dL on admission; four were in diabetic ketoacidosis.

While transient increases in blood glucose may be due to stress and more prolonged elevations can occur during treatment with corticosteroids, we found 23 of 54 (45.6%) COVID‐19 patients with preDM had persistently and markedly elevated FBG in the absence of corticosteroid therapy. Similarly, six patients without DM and with normal HbA1C levels also had repeatedly elevated FBG. Together, these 29 patients had FBG levels consistent with new onset DM and temporally associated with recent acquisition of SARS‐CoV‐2 infection. These findings support the possibility of direct dysregulation of glucose metabolism due to a newly acquired viral infection and point to an increased likelihood of developing severe COVID‐19 in these high‐risk patients.

## 4 DISCUSSION

Identifying risk factors for development of severe COVID‐19 in patients hospitalized with SARS‐2‐CoV is imperative for informing clinical decisions around patient care. Initial reports of COVID‐19 from countries impacted early in the pandemic showed a strong association between older age and risk of COVID‐19 mortality.^4^, ^5^ Data now emerging from the United States, which has the highest number of COVID‐19 cases globally to date, has demonstrated increased incidence of severe COVID‐19 in patients with co‐morbid conditions including hypertension, obesity, and diabetes. These findings have important implications for managing the clinical course of COVID‐19 in severely ill patients and further understanding the pathophysiology of this disease.

Our single‐center, consecutive series study of 184 patients with COVID‐19 demonstrates several important findings. The majority of patients who developed moderate or severe COVID‐19 had DM or preDM based on their HbA1C levels. Severe COVID‐19, defined by the need for intubation, did not occur in the absence of DM, whether pre‐existing or newly diagnosed, preDM or obesity. Our data also establish that SARS‐CoV‐2 infection significantly worsens hyperglycemia in patients with glucose metabolism problems.

Our data in patients with severe COVID‐19 and DM are consistent with a recent report by Bhatraju et al.^8^ In both studies, 58% to 62% of patients severely ill with COVID‐19 were diabetic with mean BMIs > 30 and the majority had elevated blood glucose. Additionally, we found 24% of patients with moderate‐severe COVID‐19 in our study were prediabetic. Taken together these data suggest that insulin resistance and uncontrolled glycemia play a significant role in worsening COVID‐19. In all critically ill COVID‐19 patients, blood glucose levels were elevated and tight glycemic control may therefore be an important consideration for improving clinical outcomes.

Several studies on patients with COVID‐19 have reported on diabetes as a pre‐existing diagnosis. In two recent observational studies, approximately 36% of patients with COVID‐19 were diabetic.^16^, ^17^ These studies relied on passive surveillance at the time of admission. Similarly, 42.9% of our patients were known diabetics at the time of admission. However, we specifically reviewed prior medical records to ascertain each patient's diabetes status. Further, we diagnosed an additional 17.1% during this admission. Most studies have not reported on prediabetes, a recognized syndrome with impaired glucose metabolism. By measuring HbA1C levels in every patient, we diagnosed 20.4% with prediabetes. Because of active surveillance, our data diabetes and prediabetes are replete and accurate. We have seen several nondiabetic COVID‐19 patients with persistently high FBG levels.

Binding of ACE2 by SARS‐CoV‐2 in COVID‐19 also suggests that prolonged uncontrolled hyperglycemia, and not just a history of DM, may be important in the pathogenesis of the disease.^18^ A known history of DM and ambient hyperglycemia were found to be independent risk factors for morbidity and mortality in SARS.^9^ In a follow‐up analysis of 135 patients, high fasting plasma glucose (FPG) was an independent predictor of SARS mortality.^19^ Diabetes was found in 7.4% of a cohort of hospitalized patients with COVID‐19 and appeared to be a risk factor for severity of disease.^20^ Mortality of COVID‐19 in patients with diabetes was found to be 7.6% versus 0.9% in patients with no co‐morbidities.^4^

A possible explanation for a link between hyperglycemia and ACE2 levels in the severity of COVID‐19 disease could be explained by several clinical observations in SARS and preclinical observations in the NOD diabetic mouse. Potential changes in glycosylation of the ACE2, as well as glycosylation of the viral spike protein, both possibly induced by uncontrolled hyperglycemia, may alter both the binding of the viral spike protein to ACE2 and the degree of the immune response to the virus.

In a subset of 39 patients who had no prior diabetes, received no steroid treatment during hospitalization, and who survived SARS, FPG levels during hospitalization were found to decrease before discharge.^19^ Twenty of these 39 (51%) patients had diabetes during hospitalization,^19^ and at 3 years of follow‐up only 2 out of 39 (5%) did. This suggests a mechanism of transient hyperglycemia induced by a transient inflammation of the islet cells of the pancreas by SARS‐CoV through binding of SARS‐CoV to the ACE2 present on islet cells, resulting in a transient insulin dependent DM, which resolved with resolution of disease.^19^

ACE2 protein levels in the lung of NOD diabetic mouse were elevated when compared with control mice and returned to the control level when insulin was administered.^21^ Glycemic control could reduce levels of glycosylated ACE2 in the lung, possibly reducing the number of viral binding sites, and possibly ameliorate some of the inflammation and symptoms of COVID‐19 disease. A possible paracrine loop hypothesis for COVID‐19 infection is suggested, where the virus infects the pancreas and lung, leading to hyperglycemia and upregulation of ACE2 in the lung, and further virus binding and inflammation. Poor glycemic control could therefore make the disease more severe. In a case series of 138 patients with COVID‐19, glucocorticoid therapy was used in 44.9% of non‐intensive care unit (ICU) patients and 72.2% of ICU patients,^22^ and presumably this glucocorticoid use made hyperglycemia, and possibly clinical symptoms, more severe.

Aberrently glycosylated ACE2 in the lung, nasal airways, tongue, and oropharynx in uncontrolled hyperglycemia could increase SARS‐CoV‐2 viral binding sites, thus leading to a higher propensity to COVID‐19 infection and a higher disease severity.

If true, this argues for better glycemic control in patients with pre‐diabetes and diabetes as a potential mechanism to slow COVID‐19 spread and reduce the severity of symptoms. Additionally, since 3.8% of the American population without a history of diabetes or pre‐diabetes has a HbA1C over 6.1% in random sampling,^23^ use of high A1c as a risk stratification for COVID‐19 could have merit.

Clinically, SARS‐CoV‐2 appears to cause new or worsening hyperglycemia, which may lead to more severe pneumonia. In our experience, a tipping point is reached in patients with COVID‐19 who have symptoms lasting anywhere from 2 days to over 3 weeks and the disease then “takes off.” Hospitalization before this acceleration can reduce the rate of critical illness.

It is important to note that our study has several limitations. Patients were seen at a single clinical site and cared for by one group of clinicians. While it is possible our study population is disproportionately weighted towards patients with poor underlying health, the patients with COVID‐19 in this study were consecutive referrals to our service over the course of 7 weeks in a suburban hospital. It is, therefore, unlikely that a selection bias exists, except for the criteria used by the admitting physicians. Diabetes itself was not considered a criterion for referral.

Given the urgency of finding solutions to this present crisis, our findings may assist in prognostication and triage decisions. Our data shed light on the impact of DM, preDM, and uncontrolled hyperglycemia in driving severe COVID‐19 and will facilitate identification of novel pathogenesis pathways associated with SARS‐CoV‐2 infection. This, in turn, may lead to new approaches to therapeutic intervention. Our data currently support the use of tight glycemic control in patients with hyperglycemia. Tight glycemic control was associated with a decrease in mortality (hazards ratio [HR], 0.14; CI, 0.06‐0.60; *P* = .008) as well as a decrease in ARDS (HR, 0.47; CI, 0.27‐0.83; *P* = .009) in an observational study of 500 propensity score matched patients with COVID‐19.^24^ Our observations are also in line with the WHO recommendation that corticosteroids not be used for COVID‐19 pneumonia.

Finally, our findings caution that COVID‐19 patients with DM, preDM, or obesity should be monitored closely. Those not infected should be particularly careful to avoid exposure to SARS‐CoV‐2. This information may be useful in healthcare and other settings to reduce the chances of infection in these high‐risk individuals and, conversely, to help triage nonDM, normal glycemic patients with COVID‐19 safely and efficiently.

## CONFLICT OF INTERESTS

The authors declare that there are no conflict of interests.

## AUTHOR CONTRIBUTIONS

SMS, AB, and RIC designed the study. SMS and AB performed the statistical analyses and interpretations. JAT, AB, EU, DAM, KC, and DPD collected, organized, and contributed to interpretation of the data. SMS and RIC were in charge of overall direction and planning.

## Supporting information

Supporting information
