# Supplementary material for: Impaired glucose metabolism in patients with diabetes, prediabetes, and obesity is associated with severe COVID‐19
Source: J Med Virol. 2020 Jul 17;93(1):409–15. doi: 10.1002/jmv.26227 (PMC7361926; doi:10.1002/jmv.26227)
Supplement: Supplementary file 1 — Supporting information [file JMV-93-409-s001.docx]

Smith Center Covid-19 Severity Scoring System

Table S1. Points by each category.

| Points | 1 | 0 | 1 | 2 | 3 | 4 | 5 | 6 |
| --- | --- | --- | --- | --- | --- | --- | --- | --- |
| Age (yrs) |  | 18-39 | 40-49 | 50-59 | 60-69 | 70-79 |  | >= 80 |
| Diabetes |  | None | Prediabetes |  |  | Diabetes |  |  |
| BMI | <18.5 | 18.5-24.9 | 25.0-29.9 | 25.0-29.9 | 30.0 - 34.9 | 30.0 - 34.9 | 35.0 - 39.9 | >=40 |
| A1C (%) |  | <= 5.6 | 5.7 - 6.4 | 6.5 - 7.4 | 7.5 - 9.9 | >=10 |  |  |
| 1st BG (mg/dL) |  | <=124 | 125 - 199 | 200 -299 | 300 - 399 | 400-499 | <=500 |  |

The maximum points for Age, diabetes status, BMI, A1C and initial blood glucose are 6, 4, 6, 4, and 5 respectively.

The lowest possible score = 0.

The maximum possible score = 25.

In our cohort, the lowest score = 1 and the highest = 20.

Table S1. SCCS Scores for each group, Alive & Never Intubated, Died &/or Intubated, and Total Died.

| **Total Score** | **Alive & Never Intubated** | **Died &/or Intubated** | **Died Total** |
| --- | --- | --- | --- |
| 1 | 1 | 0 | 0 |
| 2 | 0 | 0 | 0 |
| 3 | 2 | 0 | 0 |
| 4 | 4 | 0 | 0 |
| 5 | 10 | 1 | 0 |
| 6 | 4 | 0 | 0 |
| 7 | 9 | 0 | 0 |
| 8 | 11 | 3 | 3 |
| 9 | 11 | 1 | 0 |
| 10 | 5 | 4 | 3 |
| 11 | 8 | 9 | 7 |
| 12 | 11 | 8 | 5 |
| 13 | 14 | 7 | 6 |
| 14 | 6 | 7 | 5 |
| 15 | 4 | 6 | 5 |
| 16 | 5 | 4 | 3 |
| 17 | 3 | 4 | 4 |
| 18 | 3 | 1 | 1 |
| 19 | 0 | 0 | 0 |
| 20 | 0 | 3 | 2 |

Table S2. SCCS Scores for each group shown in aggregate, as total number per group with SCCS Score <= a given total.

| **Total Score** | **Alive & Never Intubated** | **Died and/or Intubated** | **Died Total** |
| --- | --- | --- | --- |
| <=1 | 1 | 0 | 0 |
| <=2 | 1 | 0 | 0 |
| <=3 | 3 | 0 | 0 |
| <=4 | 7 | 0 | 0 |
| <=5 | 17 | 1 | 0 |
| <=6 | 21 | 1 | 0 |
| <=7 | 30 | 1 | 0 |
| <=8 | 41 | 4 | 3 |
| <=9 | 52 | 5 | 3 |
| <=10 | 57 | 9 | 6 |
| <=11 | 65 | 18 | 13 |
| <=12 | 76 | 26 | 18 |
| <=13 | 90 | 33 | 24 |
| <=14 | 96 | 40 | 29 |
| <=15 | 100 | 46 | 34 |
| <=16 | 105 | 50 | 37 |
| <=17 | 108 | 54 | 41 |
| <=18 | 111 | 55 | 42 |
| <=19 | 111 | 55 | 42 |
| <=20 | 111 | 58 | 44 |
